# Effectiveness of an environmental educational program on intern dentists’ knowledge and practices regarding eco-friendly green dentistry: a quasi-experimental study

**DOI:** 10.1186/s12909-024-06523-7

**Published:** 2025-01-08

**Authors:** Eman Helmy Hassan, Nesma Lotfy, Mamdouh Hanafy Abdou, Ebtisam Mohamed Fetohy, Mohamed Fakhry Hussein

**Affiliations:** 1 Public Health Sciences, Department of Occupational Health and Industrial Medicine, Alexandria University, Alexandria, Egypt; 2 Department of Biostatistics, High Institute of Public Health, Alexandria University, Alexandria, Egypt; 3 Environmental Health, High Institute of Public Health, Alexandria University, Alexandria, Egypt; 4 Health Education and Behavioral Sciences Department, High Institute of Public Health, Alexandria University, Alexandria, Egypt; 5 Environmental Health, High Institute of Public Health, Alexandria University, Alexandria, Egypt

**Keywords:** Environmental programs, eco-friendly, green dentistry, knowledge, intern dentists, practice

## Abstract

**Background:**

The dental industry is associated with significant environmental impacts so there is a growing need for eco-friendly practices in dentistry. This study aimed to assess dental interns’ knowledge and practices regarding eco-friendly dentistry before and after the implementation of the environmental educational program.

**Methods:**

An interventional quasi-experimental study (one group pre-test-post-test design) was conducted on 69 intern dentists at the Faculty of Dentistry Alexandria University. Assessment of knowledge and practices related to eco-friendly dentistry was performed three times: before, after two months, and after four months of the educational program.

**Results:**

The mean age of participants was 23.72 years; 56.5% were males; 89.9% hadn’t received prior training on eco-friendly dentistry; and 40.6% obtained their green dentistry information from online websites. The educational program significantly improved their knowledge (median score percentage went from 50% before the intervention to 81.82% and 81.82% after two and four months of the conducting of the educational program, respectively.) and eco-friendly practices (median score percentage increased from 58.33% before the intervention to 75% and 66.67% after two and four months of the intervention, respectively) and the differences were statistically significant (*p* < 0.05). Significant positive correlations were noticed between knowledge and practice either before or after the training program (*p* < 0.05).

**Conclusions:**

There was a significant improvement in knowledge and practices of the participants after the implementation of eco-friendly educational program. The successful educational program in early career is important in reducing waste generation and resource consumption, as well as potential cost savings on the long run.

**Clinical trial registration number:**

PACTR202405544685014. Date: 20 May 2024, “retrospectively registered”.

**Supplementary Information:**

The online version contains supplementary material available at 10.1186/s12909-024-06523-7.

## Background

The dental industry has been associated with significant environmental impacts as its traditional practices include the use of non-recyclable materials, excessive water consumption, and hazardous waste generation. So, dentists have a vital role to play in protecting natural resources and reducing the environmental impact of their work. Due to the high ecological footprint of dentistry, the concept of “Going Green” is gaining traction in dental practices. Eco-friendly or environment-friendly materials minimize negative environmental effects, offering a more sustainable approach. By adopting environmentally friendly practices, dentists can achieve significant benefits. These include conserving energy and water resources, reducing waste generation, using non-toxic chemicals, promoting green products and even lowering operating costs [1–3].

The four processes that constitute most of the dental practice waste and pollution are, using conventional vacuum saliva ejector systems, conventional X-ray systems, old infection control procedures, and placing or removing dental items that contain mercury. A holistic approach to dental care known as “green dentistry”, which aims to minimize the negative effects of dentistry on the environment while also providing patients with a comfortable setting, should be taken into consideration when implementing any eco-friendly program in dentistry [2, 4, 5].

Green dentistry relies on four key principles, often referred to as the “4R”: rethink, reduce, reuse, and recycle. These concepts help dental professionals integrate sustainable practices into their everyday routines. The item ‘Rethink’ involves challenging conventional methodologies, re-evaluating established practices and finding innovative solutions to reduce the environmental impact of dental practices as using digital X-rays instead of traditional film X-rays. Reduce refers to decreasing the use of resources by using energy-efficient equipment and lighting, reducing paper usage, minimizing the use of disposable items, and using low-flow taps and toilets. The third pillar, ‘Reuse’, focuses on finding ways to reuse materials that would otherwise be discarded, such as the utilization of instruments after sterilization instead of disposing of them after a single use. The last pillar, which is ‘Recycle’, means separating and recycling waste materials, such as paper, plastics, and metals. Dental offices can implement recycling programs to ensure proper sorting and disposal of waste [6, 7].

Dental students and dentists had low knowledge about the green dentistry. Application of eco-dent programs could improve the practices of dentists to be more eco-friendly [8, 9]. Implementing eco-friendly practices in dentistry faces several obstacles, such as a lack of knowledge of these techniques among dentists, the fact that reusing and recycling dental materials can be difficult due to contamination concerns and logistical challenges, and the relatively high cost of environmentally friendly materials compared to traditional options [10].

Developing countries face even greater challenges in adopting eco-friendly dentistry. These nations often struggle with poverty, limited access to advanced technologies, and weak environmental regulations. This combination of factors makes it difficult to implement and enforce sustainable practices. Despite these challenges, integrating education on green dentistry into the early stages of a dentist’s career is crucial. By providing dentists with practical solutions alongside environmental knowledge, eco-friendly approaches will be impeded in their practices throughout their careers [11–13].

Protecting the environment and promoting well-being are important goals for dental professionals. This can be achieved through the adoption of sustainable practices and the utilization of eco-friendly materials and equipment within dental offices, thereby reducing their environmental footprint [1, 5, 7]. To achieve this, we need to bridge the knowledge gap regarding eco-friendly dentistry. This study assessed the knowledge and practices of dental interns at Alexandria University regarding green dentistry. We then developed and delivered an educational program to introduce them to these concepts. Finally, we evaluated the program’s effectiveness in improving participants’ knowledge and practices.

**The hypotheses of the study were that** intern dentists didn’t have enough knowledge about green dentistry and didn’t use many eco-friendly practices, so their participation in the educational program would significantly improve their eco-friendly green dentistry knowledge and practices.

## Methods

### Study setting and design

The study was conducted in the intern dentists’ clinics at the Faculty of Dentistry, Alexandria University, over a period of six months, from June 2023 through November 2023. The study was an interventional quasi-experimental study (one-group pre-test-post-test design).

### Target population

Intern dentists who were working at the Faculty of Dentistry Alexandria University at the time of the study were included in the study.

### Sample size

The researchers assumed a small effect size (0.2) from the education program on intern dentists’ knowledge and practice of eco-friendly dentistry. Using alpha error of 0.05 and 95% power, and number of measurements is 3, the minimum sample size required was found to be 66 interns. The sample size was calculated using G-power software. A total of 69 intern dentists who were officially registered in the Faculty of Dentistry Alexandria University were recruited in the study. The observed number of participants enrolled in the internship program for dentists was deemed sufficient for conducting a group pretest-post-test design to effectively identify any alterations in knowledge and practice.

### Data collection methods and tools

#### A pre-intervention phase

To assess dentists’ knowledge and practices related to green dentistry, we used a structured, pre-designed questionnaire (Supplementary File I). This questionnaire was adapted from existing validated questionnaires [14, 15]. To ensure its validity and clarity, content and face validity were assessed by three professors in public health, health education, and dental medicine. Based on their feedback, we added a few new questions and revised existing ones to make the questionnaire clearer. Finally, a pilot study on 10 dentists (who were not part of the main research) was conducted to test to find out the feasibility and understandability of the tool before utilizing it in the main study.

The questionnaire consisted of three main sections. The first part of the questionnaire focused on collecting personal data, including gender, age, residence, marital status, and attendance at any prior eco-friendly dentistry training programs. Additionally, participants were asked to indicate sources of their knowledge of green dentistry.

The second part pertained to the dentists’ perception and knowledge of eco-friendly dentistry by asking 18 questions. It encompassed inquiries regarding the perception of the concept of eco-friendly dentistry (two questions), and the rest of this part assessed the knowledge through asking about the four principles of going green (rethink, reduce, reuse, and recycle), major sources of dental waste and pollution, and the obstacles that hinder the implementation of such practices.

Each question was awarded points based on the answer’s accuracy. For yes-or-no questions, 1 point for a correct answer and 0 points for an incorrect one were given. In the questions with multiple answers or fill-in-the-space questions, the complete right answer had a score of “2”. For an incomplete right answer, a score of “1” was given. For the wrong answer or don’t know, a zero score was given. The knowledge questions have a total of 22 points.

The total sum of knowledge score, the mean score and the mean percentage scores were computed to evaluate the level of knowledge among intern dentists regarding eco-friendly green dental strategies. Subsequently, the knowledge was categorized into the following grades: poor knowledge (0 to less than 50%), fair knowledge (50–75%), and good knowledge (more than 75%).

The final section of the questionnaire assessed the dentists’ current eco-friendly practices through a checklist of six questions. These questions focused on everyday actions that can minimize environmental impact, such as reusing scrap paper for notes, turning off electronics when not in use, using washable lab coats, utilizing rubber dam isolation during amalgam removal, choosing alternatives to amalgam fillings, and separating waste at the source.

The answer to each question was scored as follows: score (2) for always, score (1) for sometimes, and score (0) for never. The total score (12 points) and percentage score (total score divided by the maximum possible score) were calculated for each participant. Their practices were then categorized as follows; good practice if he/she gained more than 75% of the total score, fair practice for 50–75%, poor practice for less than 50% of the total score.

#### Intervention phase

The results from the pre-intervention questionnaire helped us refine the educational program to address the areas where the intern dentists needed the most improvement. These areas included understanding the concept of eco-friendly dentistry and its benefits, recognizing the environmental impact of traditional dental practices, learning the 4R concepts of going green, Identifying the major sources of dental waste and pollution (which are the placement and removal of mercury-containing dental material, conventional x-ray systems, some infection control methods, and saliva ejector systems), understanding the barriers that hinder the implementation of eco-friendly practices, recognizing digital radiography as a sustainable alternative, learning proper waste recycling and segregation techniques, implementing energy-saving measures, using reusable lab coats, and exploring alternatives to amalgam fillings.

The 69 participating dentists were divided into three groups. Each group received a three-week educational program consisting of three sessions, each lasting 45–60 min and spaced one week apart. The sessions used a variety of engaging methods to deliver the information, such as clear and concise PowerPoint presentations, which helped explain key concepts, and informative videos that supported the learning process. Also, group discussion and brainstorming were implemented throughout the educational program. To reinforce the learning, posters summarizing key points from the program were displayed in the dental clinics.

#### Post-intervention phase

To evaluate how the educational program influenced the dentists’ knowledge and practices, we administered the same questionnaire again two and four months after they completed the program. This follow-up helped us assess the program’s effectiveness in promoting eco-friendly dentistry in dental offices.

### Pilot study

The pilot study was carried out on a group of 10 intern dentists. The group in the pilot study was not a part of the main study sample. The pilot study aimed at testing the feasibility of collecting data and understandability of the questionnaire as well as recognizing obstacles that could be met during the execution of the research. It is also used to estimate the time needed to complete the questionnaire, which was found to be 10 to 15 min. A few changes were made to clarify the meaning of some sentences.

### Ethical considerations

The Higher Institute of Public Health’s Ethics Committee, Alexandria University, gave the researchers permission to perform the study (IRB number: 00013692). The clinical trial registration number is PACTR202405544685014. The International Guidelines for Research Ethics were followed by the researcher. Following an explanation of the goals and advantages of the study, each participant gave their informed permission. The subjects’ involvement was entirely voluntary, and they were able to leave the research at any moment. Confidentiality and anonymity were guaranteed and upheld.

### Statistical analysis

The information that was gathered was introduced and analyzed with IBM SPSS software package version 25.0. (Armonk, NY: IBM Corp). For categorical data, frequencies and percentages were utilized. The Shapiro-Wilk test was utilized to investigate the normality of data. The data doesn’t follow the normal distribution so the median and range (Min-Max) was employed for continuous variables. The Chocharn’s Q test was used to determine if there are differences on a dichotomous dependent variable between three or more measurements. If the Q test is significant, a post hoc (adjusted Bonferroni) was conducted to determine the significance of the pair of measurements. Marginal homogeneity, which is used to pair nominal data with more than 2 categories. The Friedman test was used to assess the difference between the three measurements of the total score of knowledge and practice. If the Friedman test is significant a post hoc (adjusted Bonferroni) was conducted to determine the significance of pair of measurement. Correlation between knowledge and practice was assessed by Spearman’s rank correlation coefficient test (rho). Relation between socio-demographic data and knowledge and practice levels was performed by Mann-Whitney test. If the p-value for an inferential analysis was less than 0.05, it was considered statistically significant.

## Results

Table 1 shows some socio-demographic features of the participants. The study recruited 69 intern dentists, whose ages ranged from 22 to 27 years, with a mean age of 23.72 ± 1.027 years. More than half of the participants were males (56.5%). All of them were residents of Alexandria. Regarding knowledge about green dentistry, the vast majority did not attend a training program about eco-friendly dentistry (89.9%).

**Table 1:** Socio-demographic characteristics of the participants and source of eco-friendly dental knowledge

Background information and source of knowledge	*n* = 69	%
Age (years) • Mean ± SD • Minimum-Maximum • Median	23.72 ± 1.027 22–27 24	
Gender • Male • Female	39 30	56.5 43.5
**Attendance of training program for eco-friendly dentistry** • No • Yes	62 7	89.9 10.1

Table 2 provides an overview of the overall knowledge score concerning eco-friendly dentistry among the intern dentists under study. After comparing the proportion of accurate responses about knowledge of eco-friendly green dentistry prior to and after two months following educational intervention, there was a noteworthy rise in knowledge across all inquiries (*p* < 0.05), except in Q11, Q12, Q16, and Q17 (*p* > 0.05). There were no significant variations (*p* > 0.05) observed between the correct responses obtained after 4 months following the educational intervention and those taken two months after, except in Q3 (*p* = 0.02) and Q4 (*p* = 0.009).

Regarding overall knowledge scores, before the intervention, only 7.2% of the intern dentists possessed good knowledge of eco-friendly dentistry. Two months after the intervention, there was a significant improvement, with 72.5% of the intern dentists exhibiting good knowledge. It is worth noting that during the four-month post-test, the participants maintained their high knowledge level, with 69.6% of intern dentists demonstrating good knowledge. A statistically significant difference was observed between the pre-intervention and post-intervention results (*p* < 0.001). The non-significant difference between the two- and four-month post-intervention results indicated good retention of the information. (Table 2)

**Table 2:** Total knowledge score towards eco–friendly dentistry among the studied intern dentists before and after the educational program

Dentists’ knowledge towards eco-friendly dentistry	Pre-intervention (*n* = 69)		After 2 months of the intervention (*n* = 69)		After 4 months of the intervention (*n* = 69)		*p*	Post hoc test		
	No.	%	No.	%	No.	%		p1	p2	p3
Q1. Hearing about eco-friendly dentistry (EFD) concept										
No Yes	54 15	78.3 21.7	8 61	11.6 88.4	4 65	5.8 94.2	**P**^**# =**^ 0.000	< 0.001	< 0.001	0.999
Q2. Hearing about Eco-Friendly Dentistry Association										
No Yes	61 8	88.4 11.6	17 52	24.6 75.4	16 53	23.2 76.8	**P**^**# =**^ 0.000	< 0.001	< 0.001	0.999
Q3. The concept of eco-friendly dentistry reduces the environmental impact of dental practices										
Don’t Know Wrong answer Incomplete right answer Complete right answer	39 1 1 28	56.5 1.4 1.4 40.6	0 2 3 64	0 2.9 4.3 92.8	6 1 7 55	8.7 1.4 10.1 79.7		< 0.001	< 0.001	0.02
Q4. Dental practices can have a negative impact on the environment.										
Don’t know No Yes	15 4 50	21.7 5.8 72.5	2 0 67	2.9 0 97.1	7 5 57	10.1 7.2 82.6		< 0.001	0.071	0.009
Q5. Knowing the effects of dental practices on the environment.										
Non-applicable Non Incomplete right answer Complete right answer	11 2 30 26	15.9 2.9 43.5 37.7	2 0 13 54	2.9 0 18.8 78.3	3 1 13 52	4.3 1.4 18.8 75.4		< 0.001	< 0.001	0.515
Q6. Feeling responsible for avoiding harm to the environment										
No Yes	11 58	15.9 84.1	2 67	2.9 97.1	6 63	8.7 91.3	**P**^**# =**^ 0.017	0.013	0.342	0.618
Q7. Knowing how to reduce the harm produced to the environment by dentistry										
Non-applicable No Yes	3 41 25	4.3 59.4 36.2	0 5 64	0 7.2 92.8	1 7 61	1.4 10.1 88.4		< 0.001	< 0.001	0.317
Q8. Knowing that green dentistry depends on 4 main steps named 4Rs.										
No Yes	62 7	89.9 10.1	13 56	18.8 81.2	11 58	15.9 84.1	**P**^**# =**^ 0.000	< 0.001	< 0.001	0.999
Q9. Knowing that the 4 steps of going green are Reduce, Reuse, Recycle, Rethink.										
Don’t know wrong Incomplete right answer Complete right answer	32 2 3 32	46.4 2.9 4.3 46.4	5 2 2 60	7.2 2.9 2.9 87.0	10 1 5 53	14.5 1.4 7.2 76.9		< 0.001	< 0.001	0.077
Q10. Mercury-containing dental materials are a significant source of pollution in dental practices.										
No Yes	9 60	13.0 87.0	0 69	0.0 100.0	3 66	4.3 95.7	**P**^**# =**^ 0.003	0.003	0.080	0.804
Q11. Conventional X-ray systems contribute significantly to dental practice waste and pollution.										
No Yes	13 56	18.8 81.2	4 65	5.8 94.2	8 61	11.6 88.4	**P**^**# =**^ 0.079			
Q12. Infection control methods can generate significant waste and pollution in dental practices.										
No Yes	16 53	23.2 76.8	7 62	10.1 89.9	12 57	17.4 82.6	**P**^**# =**^ 0.07			
Q13. Conventional vacuum saliva ejector systems can contribute to dental practice waste and pollution.										
No Yes	17 52	24.6 75.4	6 63	8.7 91.3	8 61	11.6 88.4	**P**^**# =**^ 0.009	0.012	0.056	0.999
Q14. It is acceptable to dispose fixer solution down the drain										
Don’t know No Yes	24 37 8	34.8 53.6 11.6	6 48 15	8.7 69.6 21.7	9 39 21	13.0 56.5 30.5		0.002	< 0.001	0.674
Q15. Used X-ray film is considered hazardous waste.										
Don’t know No Yes	17 10 42	24.6 14.5 60.9	5 3 61	7.3 4.3 88.4	8 4 57	11.6 5.8 82.6		< 0.001	0.009	0.286
Q16. Digital dental radiography creates waste.										
Don’t know No Yes	7 44 18	10.1 63.8 26.1	3 57 9	4.4 82.6 13.0	9 39 21	13.0 56.5 30.5		0.398	0.876	0.303
Q17. It is acceptable to use reusable stainless-steel cups, reusable stainless steel surgical/ endodontic suction tips and glass syringes as endodontic irrigation devices after their sterilization.										
Don’t Know No Yes	10 16 43	14.5 23.2 62.3	6 11 52	8.7 15.9 75.4	12 8 49	17.4 11.6 71.0		0.074	0.642	0.216
Q18. Knowing the barriers that prevent application of eco-friendly dental practices										
Non-applicable Incomplete right answer Complete right answer	17 45 7	24.6 65.2 10.2	8 47 14	11.6 68.1 20.3	9 49 11	13.1 71.0 15.9		0.004	0.006	0.484
**Overall knowledge** Poor knowledge (< 50%) Fair knowledge (50–75%) Good knowledge (> 75%)	29 35 5	42.0 50.8 7.2	1 18 50	1.4 26.1 72.5	5 16 48	7.2 23.2 69.6	**p**^**† =**^ <0.001*	< 0.001*	< 0.001*	0.999
**Score percentage** Min – Max Median	0.0– 81.82 50		40.91–95.45 81.82		13.64–95.45 81.82					

P^#^ = Q test

p^†^: Friedman test

Marginal homogeneity test was used for each pair for q3,4,5,7,9,14,15,16,17,18

p1: p-value for comparing between pre-intervention and after 2 months of the intervention

p2: p-value for comparing between pre-intervention and after 4 months of the intervention

p3: p-value for comparing between after 2 months of the intervention and after 4 months of the intervention

* Statistically significant at *p* < 0.001

Table 3 illustrates how the educational program improved the intern dentists’ eco-friendly practices. After comparing the proportion of accurate responses about practice of eco-friendly green dentistry prior to and after two months following educational intervention, there was not a rise in practice across all inquiries (*p* > 0.05), except in Q1 (*p* < 0.001) and Q4 (*p* = 0.007).

Regarding overall practice score, before the program, only 21.7% of dentists had good eco-friendly practices. After the program, these scores increased significantly up to53.6% at two months and 46.4% at four months (*p* < 0.05). The median score percentage increased also from 58.33 to 75% and 66.67% after two and four months of the intervention, respectively.

**Table 3:** Total score of intern dentists’ practices regarding eco-friendly dentistry before and after the educational program

Dentists’ practice towards eco-friendly dentistry	Pre-intervention (*n* = 69)		After 2 months of the intervention (*n* = 69)		After 4 months of the intervention (*n* = 69)		*p*	Post hoc test		
	No.	%	No.	%	No.	%		p1	p2	p3
Q1. Reuse of previously utilized papers derived from scratch pads or internal notes.										
Never Sometimes Always	46 20 3	66.7 29.0 4.3	29 29 11	42.0 42.0 16.0	25 27 17	36.2 39.2 24.6		< 0.001	< 0.001	0.232
Q2. Switch off electronic devices when they are not in use.										
Never Sometimes Always	10 21 38	14.5 30.4 55.1	10 14 45	14.5 20.3 65.2	9 24 36	13.0 34.8 52.2		0.297	0.884	0.302
Q3. Using lab coats that can be laundered.										
Never Sometimes Always	9 10 50	13.0 14.5 72.5	8 6 55	11.6 8.7 79.7	7 10 52	10.1 14.5 75.4		0.473	0.593	0.808
Q4. Using rubber dams during removal of old amalgam to reduce the vapor released.										
Never Sometimes Always	25 23 21	36.3 33.3 30.4	14 21 34	20.3 30.4 49.3	17 21 31	24.6 30.5 44.9		0.007	0.047	0.491
Q5. Utilization of alternatives to amalgam filling.										
Never Sometimes Always	6 13 50	8.7 18.8 72.5	6 13 50	8.7 18.8 72.5	9 15 45	13.0 21.7 65.3		0.999	0.217	0.258
Q6. Segregation of waste at the point of origin.										
Never Sometimes Always	18 23 28	26.1 33.3 40.6	10 27 32	14.5 39.1 46.4	5 22 42	7.2 31.9 60.9		0.128	0.001	0.039
**Overall practice** Poor practice (< 50%) Fair practice (50–75%) Good practice (> 75%)	11 43 15	15.9 62.4 21.7	10 22 37	14.5 31.9 53.6	6 31 32	8.7 44.9 46.4	**p**^**†=**^ 0.004*	0.028*	0.017*	0.999
**Score percentage** Min – Max Median	0.0 – 91.67 58.33		0.0–100 75		25–100 66.67					

p^†^: Friedman test

Marginal homogeneity test was used for each pair for all questions

p1: p-value for comparing between pre-intervention and after 2 months of the intervention

p2: p-value for comparing between pre-intervention and after 4 months of the intervention

p3: p-value for comparing between after 2 months of the intervention and after 4 months of the intervention

* Statistically significant at *p* < 0.001

Table 4 shows the correlation between knowledge and practice of green dentistry among intern dentists. A significant positive correlation was found between knowledge and practice before the educational program (*p* < 0.001). This significant positive correlation was maintained after two and four months of the training (*p* < 0.001 and *p* < 0.05, respectively).

**Table 4:** Correlation between knowledge and practice among intern dentists before and after the educational program

Correlation between knowledge and practice	Spearman’s correlation coefficient test (rho)	*p*-value
**Before program**	0.528**	< 0.001
**After 2 months**	0.419**	< 0.001
**After 4 months**	0.259*	0.032

*p-value significant at < 0.05 **p-value significant at < 0.001

Table 5 illustrates the relation between socio-demographic characteristics and previous attendance at training programs about eco-friendly dentistry and total knowledge and practice scores across time. No significant differences were found when comparing the scores of knowledge and practice to the categories of each socio-demographic variable either before or after the program (*p* > 0.05). However, significant improvement in knowledge could be observed immediately after two months of the educational intervention in all variables considered in the study (*p* < 0.05). In addition, significant differences were revealed when comparing practice scores across time for 22-<24 age, female, and who didn’t attend previous training programs for eco-friendly dentistry groups (*p* < 0.05).

**Table 5:** The relation between socio-demographic characteristics and previous attendance of training and knowledge and practice score before and after the educational program

Variable	Category		Pre-intervention				After 2 months of the intervention				After 4 months of the intervention				*p* ^†^		Post-hoc				
			Median (Min-Max)		*P* ^#^		Median (Min-Max)		*P* ^#^		Median (Min-Max)		*P* ^#^				p1		p2		p3
**Knowledge**																					
**Age**	**22-**		11(6–18)		0.818		18(11–20)		0.374		18(7–21)		0.538		< 0.001*		< 0.001*		< 0.001*		0.999
	**24-**		11(0–17)				18(9–21)				18(3–21)				< 0.001*		< 0.001*		< 0.001*		0.754
**Gender**	**Male**		12(6–17)		0.197		18(11–20)		0.346		18(3–21)		0.578		< 0.001*		< 0.001*		< 0.001*		0.999
	**Female**		11(0–18)				18.5(9–21)				18(9–21)				< 0.001*		< 0.001*		< 0.001*		0.526
**Previous attendance of training program for eco-friendly dentistry**	**No**		11(0–18)		0.503		18(9–21)		0.293		18(3–21)		0.802		< 0.001*		< 0.001*		< 0.001*		0.999
	**Yes**		12(6–16)				19(16–21)				18(7–21)				0.002*		0.048*		0.004*		0.99
**Practice**																					
**Age**		**22-**		7(1–10)		0.079		9(0–12)		0.855		8(3–12)		0.999		0.005*		0.047*		0.019*	0.999
		**24-**		8(0–11)				8.5(0–12)				8(5–12)				0.278					
**Gender**		**Male**		8(1–11)		0.116		9(0–12)		0.308		8(3–12)		0.956		0.216					
		**Female**		7(0–10)				8(0–12)				8.5(4–12)				0.007*		0.085		0.014*	0.999
**Previous attendance of training program for eco-friendly dentistry**		**No**		7(0–11)		0.529		9(0–12)		0.27		8(3–12)		0.273		0.004*		0.059		0.011*	0.999
		**Yes**		8(1–10)				10(7–12)				6(5–10)				0.354					

P^#^: Mann-Whitney test

p^†^: Fridman test

p1: p-value for comparing between pre-intervention and after 2 months of the intervention

p2: p-value for comparing between pre-intervention and after 4 months of the intervention

p3: p-value for comparing between after 2 months of the intervention and after 4 months of the intervention

* Statistically significant at *p* < 0.05

## Discussion

The current study aimed to evaluate the impact of an eco-friendly educational program on 69 intern dentists at the Faculty of Dentistry, Alexandria University. The research used an interventional quasi-experimental design, employing pre-test and post-test interventions. Results showed that 26.1% of the participants had never heard about eco-friendly dentistry, and only 7.2% had a good knowledge level about it before the implementation of the educational program. This indicates an inadequate level of environmental consciousness. After the conduct of the educational program, there was a notable improvement in their knowledge, as 72.5% of them had a good knowledge level after two months of the program, and 69.6% of them still retained a good level of knowledge after four months of the intervention.

Many studies supported these findings as they reported that a large proportion of the professional and student dentists were not aware of green dentistry, and this knowledge gab was the main reason for non-ecofriendly behavior between them. Most students believed that eco-friendly practices were important in their future work and that they were interested in learning them. A number of obstacles to increasing knowledge about green dentistry were mentioned, including limited time, a condensed curriculum, and inadequate educational resources [16–18]. A study carried out in north India documented that 89% of intern dentists lacked knowledge of proper procedures for managing biomedical waste. On the other hand, about half of the dentists surveyed had some knowledge about recycling or reusing dental materials [19].

To see the effect of educational programs on improving green dentistry knowledge, a study conducted in Saudi Arabia showed similar results as ours, as dentists’ knowledge of eco-dentistry significantly improved after attending educational lectures (*p* < 0.05). This highlights the importance of ongoing education in promoting sustainable practices [20].

In the current study, significant positive correlations were observed between knowledge and practice before and after the intervention which means that those with good knowledge had good practices and vice versa. Similarly, Ladia and Gupta (2017) reported a positive correlation between knowledge and practice regarding dentists’ infection control and waste management [21]. On the other hand, some studies found a good level of knowledge of environmentally friendly concepts; however the practice of dentists was inadequate [14, 22–24].

While the overall practice of the intern dentists in our study was low, some eco-friendly practices were observed. The present study showed that over half (55.1%) of the dentists turned off electronic devices when not in use, which could be due to a growing knowledge of energy conservation. Additionally, about three-quarters (72.5%) preferred reusable lab coats. These findings partially align with an Indian study that documented that 89% of dentists used reusable lab coats and a significant majority (96%) powering down electronics when not in use [1].

After the implementation of the educational program, significant improvement in green dentistry knowledge and practices was observed in the current study, as about one-fifth (21.7%) of the intern dentists had good eco-friendly practices before the educational program, and a significant improvement was detected after the intervention (53.6% and 46.4% of the participants had good practices after two and four months, respectively). These results were supported by other studies, which have also shown that training programs could improve eco-friendly knowledge and practice among dentists in many topics such as infection control and waste management. However, this information was not retained after a period of time among a recognizable number of dentists [21, 25].

In the present study, younger dentists, females, and those without previous knowledge about green dentistry achieved more improvement in their practices than other categories. Younger age may be more acceptable for changing practices before establishing solid practice and may have more attention to improve their practice [26, 27]. Some studies suggest women may be more intrinsically motivated by values like social responsibility and environmental well-being. Women may already have a stronger interest in environmental issues, making them more receptive to the program’s message of sustainability [28, 29]. Individuals lacking prior knowledge may experience a steeper learning curve and show greater improvement after training sessions compared to those who attended previous programs. Dentists who hadn’t attended earlier training may have been more receptive to new information due to a lack of established habits. Perhaps the new program was designed to be more impactful than the previous ones [26, 27].

Green dentistry focuses on using eco-friendly technologies and creating a healthy work environment for both patients and staff. This approach helps to minimize the environmental impact of dental practices. Common areas of interest concerning eco-friendly dentistry practices include the management of amalgam fillings, radiographic management, infection control procedures, waste disposal, energy efficiency, and water conservation [24, 30]. Our study identified a noticeable gap in intern dentists’ knowledge and practices regarding eco-friendly dentistry. However, the educational program significantly improved these items. These findings highlight the urgent need to incorporate eco-friendly dentistry principles into dental education curricula. In countries with limited resources, like Egypt, adopting eco-friendly dental practices can be difficult. The current research provides helpful ways to make dental care more sustainable, even in these challenging conditions. Equipping new dentists with this knowledge from the start of their careers will encourage them to adopt sustainable practices throughout their professional lives.

### Limitations and strength of the study

The study has some limitations. First, due to logistical constraints, the study was only conducted in one setting. Ideally, the program’s effectiveness should be tested in a wider range of dental practice environments. Second, the follow-up period only lasted four months. A longer study would be needed to see if the dentists retain the knowledge and continue the eco-friendly practices they learned. Third, while the study compared pre- and post-intervention knowledge and practices, a stronger design would involve a control group that did not receive the educational program.

Despite these limitations, the study has several points of strength. First, it had a high retention rate as no participants dropped out of the study (low attrition bias); suggesting similar programs could be successful in the future. Second, the study assessed the practice of dentists using an observational checklist. Third, this is one of the first studies to examine how an educational program can influence the eco-friendly behavior of early-career dentists throughout their careers. The study addresses a relatively under-explored area in dentistry: the importance of sustainable practices. It is one of the first studies talking about eco-friendly dentistry in Egypt. The study had a comprehensive evaluation of the program’s effectiveness by assessing both knowledge and practices.

## Conclusions

Before the educational program, the intern dentists demonstrated a limited understanding of green dentistry concepts and their practices was lacking. However, after participating in the program, their knowledge and practices improved significantly. This improvement was sustained during the follow-up visits, suggesting the dentists retained the information and began integrating eco-friendly concepts into their daily routines. Significant positive correlations were noticed between knowledge and practice either before or after the training program. Females, younger age group, and those who did not attend previous green dentistry workshops gained more benefit from our program in eco-friendly practices than other categories. The study highlights the potential of implementing eco-friendly practices in dentistry to reduce waste generation and environmental pollution.

## Electronic Supplementary Material

Below is the link to the electronic supplementary material.

Supplementary Material 1

## Data Availability

The datasets used and/or analyzed during the current study are available from the corresponding author upon reasonable request.

## Abbreviations

P: Probability value
Rho: Spearman’s rank correlation coefficient test
SD: Standard Deviation
4R: Rethink, Reduce, Re-use, and Recycle
