# Supplementary material for: Effectiveness of an environmental educational program on intern dentists’ knowledge and practices regarding eco-friendly green dentistry: a quasi-experimental study
Source: BMC Med Educ. 2025 Jan 8;25:32. doi: 10.1186/s12909-024-06523-7 (PMC11714915; doi:10.1186/s12909-024-06523-7)
Supplement: Supplementary file 1 — Supplementary Material 1 [file 12909_2024_6523_MOESM1_ESM.docx]

**Supplementary File I**

**Questionnaire**

**Full Name:**

**Serial Number:**

**Dear intern dentists:**

We would like to invite you to participate in a research study aimed at improving the environment. The purpose of this questionnaire is to gather information about your experiences and perceptions of eco-friendly green dentistry, and your feedback will be used to make improvements that benefit patients, staff and the environment.

We want to assure you that your name and personal information will not be used in any way that could identify you. Your responses will be assigned as code numbers for analysis purposes only.

We appreciate your willingness to help us in this important endeavor, and we believe that your input will make a significant contribution to the improvement of dental clinics.

Please take your time to answer this questionnaire. You can put more than one answer. We are trying to assess your knowledge, attitude, and practices towards eco-friendly dental practices.

**I- Background information and source of knowledge:**

1- Age: …………

2- Gender:

1. Male
2. Female

3- Residence:

1. Inside Alexandria
2. Outside Alexandria

4- Did you attend any previous training program for eco-friendly dentistry?

1. No
2. Yes

5- What is your source of knowledge concerning eco-friendly dentistry? “You can choose more than one answer”

1. Under-graduate curriculum
2. Training program
3. Mass media
4. Internet
5. Others (specify…………………………………………….)

(-1) I did not hear about it before

**II-Dentists' awareness and knowledge towards eco-friendly dental practices:**

1- Did you hear about Eco-Friendly Dentistry (EFD) concept?

- No
- Yes

2- Did you hear about Eco-Friendly Dentistry Association?

- No
- Yes

3- The concept of eco-friendly dentistry is ……..

4- Does practicing dentistry cause any environmental harm?

- No
- Don't know
- Yes

5- What are the negative effects of dentistry on the environment? “You can choose more than one answer”

- Water pollution.
- Wasting water.
- Increased burden on landfills.
- Soil pollution
- Others (specify): … … … … … … … … … … … …
- All of the above
- None of the above
- Don't know

6- It is my responsibility to avoid harming the environment.

- No
- Yes
- Don’t know

7- I know how to reduce the environmental impact of my dentistry work.

- No
- Yes

8- Green dentistry depends on 4 main steps named 4Rs.

- No
- Yes
- Don't know

9- The 4 steps of going green are ……

10- One of the main sources of dental practice waste and pollution is the placement and removal of mercury-containing dental material.

- No
- Yes
- Don't know

11- One of the main sources of dental practice waste and pollution is conventional x-ray systems.

- No
- Yes
- Don't know

12- One of the main sources of dental practice waste and pollution is infection control methods.

- No
- Yes
- Don't know

13- One of the main sources of dental practice waste and pollution is the conventional vacuum saliva ejector system.

- No
- Yes
- Don't know

14- Is it acceptable to dispose fixer solution down the drain?

- No
- Yes
- Don't know

15- Can we consider used x-ray film a hazardous waste?

- No
- Yes
- Don't know

16- Does digital dental radiography create waste?

- No
- Yes
- Don't know

17- Is it acceptable to use reusable stainless-steel cups, reusable stainless steel surgical/ endodontic suction tips and glass syringes as endodontic irrigation devices after their sterilization?

- No
- Yes
- Don't know

18- What are the barriers that prevent application of eco-friendly dental practices? “You can choose more than one answer”

- Lack of interest
- Lack of information
- High costs
- Lack of supervision
- There are no policies to regulate such practices
- Non availability of biocompatible materials
- Others (specify …………………………….)
- Don’t know

**III) Checklist for dentists' practice towards eco-friendly dentistry:**

1- Reuse of previously utilized papers derived from scratch pads or internal notes.

- Never
- Sometimes
- Always

2- Switch off electronic devices when they are not in use.

- Never
- Sometimes
- Always

3- Using lab coats that can be laundered.

- Never
- Sometimes
- Always

4- Using rubber dams during removal of old amalgam to reduce the vapor released.

- Never
- Sometimes
- Always

5- Utilization of alternatives to amalgam filling.

- No
- Sometimes
- Always

6- Segregation of waste at the point of origin.

- Never
- Sometimes
- Always

Thanks for your time
